# Supplementary material for: Genome-Wide Association Study of Body Conformation Traits in Tashi Goats (Capra hircus)
Source: Animals (Basel). 2024 Apr 9;14(8):1145. doi: 10.3390/ani14081145 (PMC11047570; doi:10.3390/ani14081145)
Supplement: Supplementary file 1 [file animals-14-01145-s001.zip › Supplementary Figures 1-3.pdf]

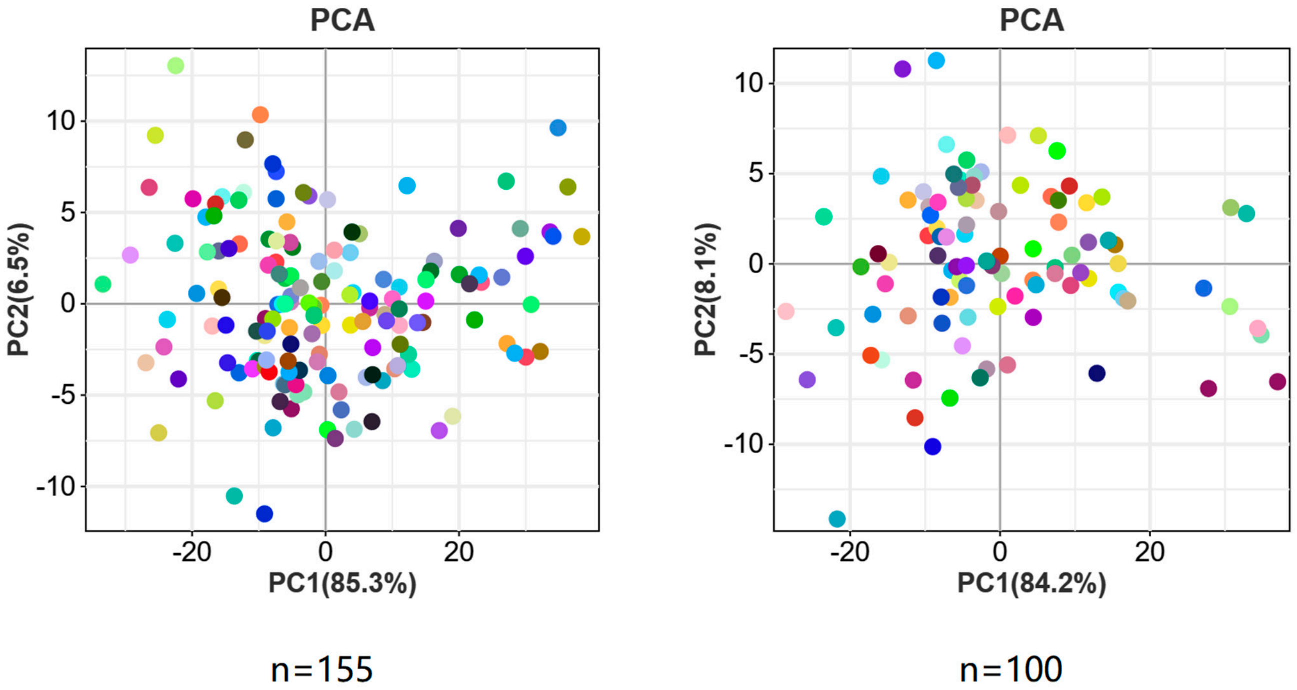

Figure S1. The PCA score plots based on phenotypical data

(A) The 155 Tashi goats; (B) The 100 Tashi goats used in genome-wide sequencing;

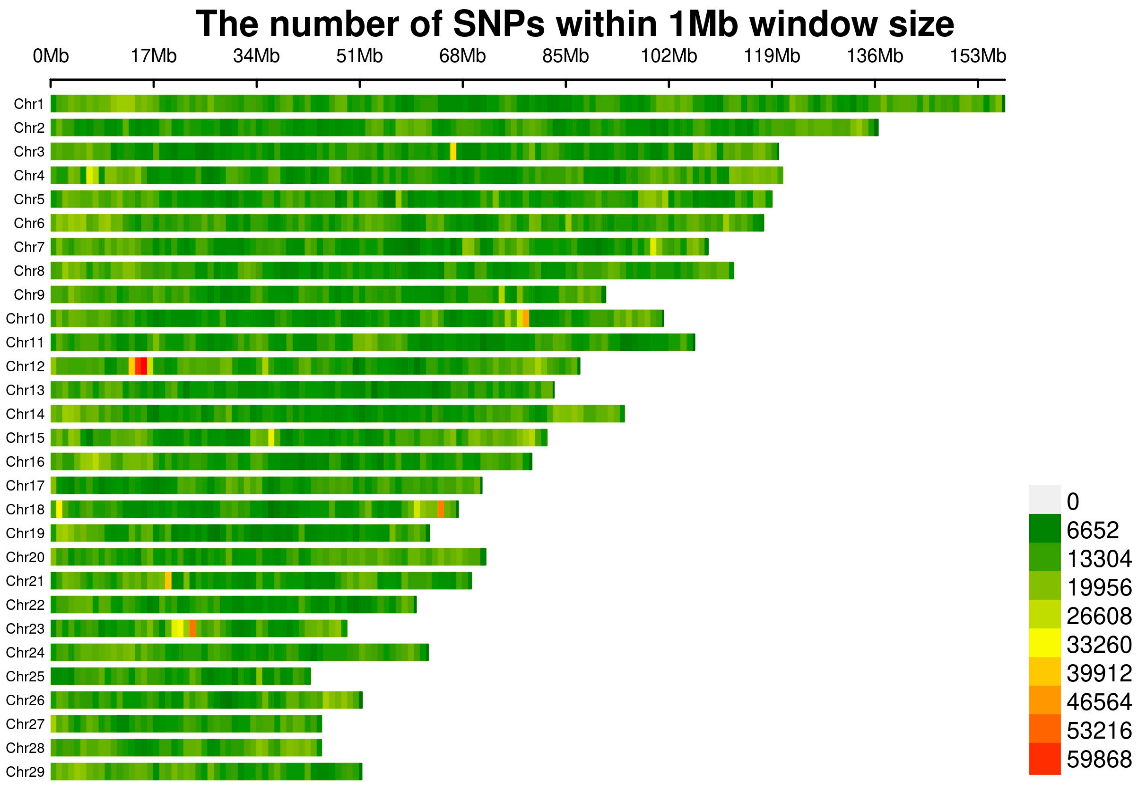

Figure S2. Distribution of SNP density throughout the genome.

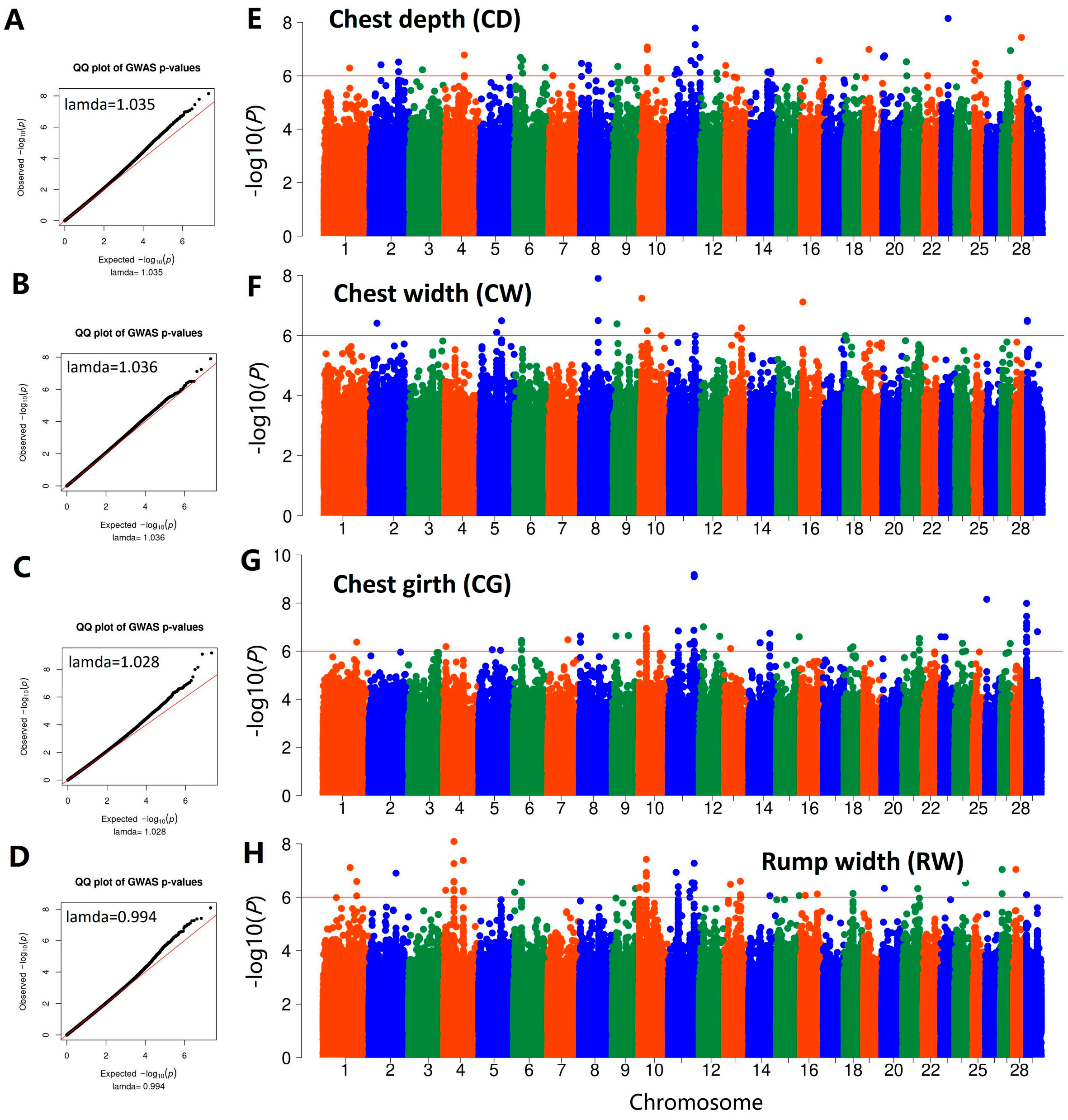

Figure S3. The Manhattan plots of four body conformation traits in Tashi goats

The QQ plots of the GWAS results for Chest depth (A), Chest width (B), Chest girth (C), and Rump width (D); The Manhattan plots of GWAS results for Chest depth (E), Chest width (F), Chest girth (G), and Rump width (H).
